# Adhesion of Triple-Negative Breast Cancer Cells under Fluorescent and Soft X-ray Contact Microscopy

**DOI:** 10.3390/ijms22147279

**Published:** 2021-07-06

**Authors:** Paulina Natalia Osuchowska, Przemysław Wachulak, Wiktoria Kasprzycka, Agata Nowak-Stępniowska, Maciej Wakuła, Andrzej Bartnik, Henryk Fiedorowicz, Elżbieta Anna Trafny

**Affiliations:** 1 Biomedical Engineering Centre, Institute of Optoelectronics, Military University of Technology, Kaliskiego 2, 00-908 Warsaw, Poland; paulina.osuchowska@wat.edu.pl (P.N.O.); wiktoria.kasprzycka@wat.edu.pl (W.K.); agata.nowak@wat.edu.pl (A.N.-S.); 2 Laser Technology Division, Institute of Optoelectronics, Military University of Technology, Kaliskiego 2, 00-908 Warsaw, Poland; przemyslaw.wachulak@wat.edu.pl (P.W.); andrzej.bartnik@wat.edu.pl (A.B.); 3 Maria Sklodowska-Curie National Research Institute of Oncology, Roentgena 5, 02-781 Warsaw, Poland; maciej.wakula@pib-nio.pl (M.W.); henryk.fiedorowicz@wat.edu.pl (H.F.)

**Keywords:** focal adhesion, adhesion, cancer cells, soft X-ray contact microscopy

## Abstract

Understanding cancer cell adhesion could help to diminish tumor progression and metastasis. Adhesion mechanisms are currently the main therapeutic target of TNBC-resistant cells. This work shows the distribution and size of adhesive complexes determined with a common fluorescence microscopy technique and soft X-ray contact microscopy (SXCM). The results presented here demonstrate the potential of applying SXCM for imaging cell protrusions with high resolution when the cells are still alive in a physiological buffer. The possibility to observe the internal components of cells at a pristine and hydrated state with nanometer resolution distinguishes SXCM from the other more commonly used techniques for cell imaging. Thus, SXCM can be a promising technique for investigating the adhesion and organization of the actin cytoskeleton in cancer cells.

## 1. Introduction

Migrating metastasis cancer cells in circulation are rare and may become resistant to chemotherapy and radiotherapy when they reach target organs [1]. Triple-negative breast cancer (TNBC) is an aggressive cancer subtype, occurring in 15–20% of breast cancer patients, with a poor prognosis due to the lack of targeted therapies [2,3,4]. The molecular mechanism of metastasis is complicated and still extensively studied in oncology and biomedical sciences. The cancer cell settlement in secondary metastatic tissues is a critical step in this process and occurs via adhesion to the extracellular matrix (ECM) [1]. Therefore, the knowledge of TNBC cell adhesion and migration mechanisms is of clinical importance [5].

Several models of cancer cell migration have already been described. Lymphoid, melanoma, and lung cancer cells with mainly cortical actin cytoskeletons migrate with weak association to ECM proteins and exhibit an amoeboid type of migration (the cells maintain rounded shape) [6,7]. In contrast, HCC38 breast cancer cells exhibit elongated shapes (the cells are polarized) and show the mesenchymal type of migration, characterized by highly developed actin stress fibers [8,9,10]. In the latter type of migration, cells adhere strongly to the ECM and migrate along the structural network provided by the ECM. The polarization of the cells on their ridges occurs to maximize the area of focal contacts. The adhesion proteins comprise larger structures, i.e., focal adhesions (FAs), intermediary elements in the cell-to-substrate connections. Targeting FAs proteins may provide an effective way to control the metastasis of TNBC cells and has recently become one of the main topics in cancer studies [1,11].

Cell adhesion and migration involve the actin cytoskeleton through its interactions with the cell membrane and the ECM. The actin cytoskeleton is linked to integrin heterodimers and other adhesion proteins that comprise FAs structures [12,13]. The forces generated by the actin cytoskeleton drive the movement process by pushing the protrusions. This process demands fast and efficient actin polymerization [14]. Actin polymerization and myosin II activity propel the leading edge of the cell. The filopodium, a finger-like protrusion, and lamellipodia, the lamellar-like protrusions, are formed. As the lamellipodium extends, promoted by actin polymerization, initial focal adhesions engage the underlying matrix [15]. Both stress fibers and focal adhesions participate in cell movement. The tearing of the stress fibers is accompanied by the rapid disassembly of the focal adhesions. When cell attachment sites break down during cell migration, stress fibers slowly weaken, either as a result of interaction with microtubules or downregulation of specific components of focal adhesion [16].

The actin cytoskeleton has a quite complex structure that comprises actin bundles and actin networks, governing different processes within the cell. Cross-linked actin filaments form bundles while loosely organized actin with the properties of semi-solid gels has a three-dimensional structure [17]. The cross-linked actin bundles arrange into more ordered structures, stress fibers (SF). There are three types of SF in the cell: ventral, dorsal, and transverse arcs. They differ in function, composition, and cellular location. Dorsal stress fibers are non-contractile and long linear structures, cross-linked by α-actinin, and anchored to FAs on the one side of the fibers that occur near the cell edges. The ventral stress fibers are also linear structures but extend most often through the entire length of the cell. They are anchored on both sides of the cell by FAs complexes. Dorsal stress fibers comprise α-actinin and trans-myosin II and generate contractile forces in the cell. The FA-anchored ventral stress fibers generate forces by interacting with the ECM. FA’s lack of anchorage in this type of SF and contractility of myosin II causes slide centripetally along the dorsal stress fibers [16].

The organization of actin cytoskeleton and adhesion proteins in cell protrusions have already been characterized with confocal microscopy (CLSM) [18,19], electron microscopy (EM) [20,21], and atomic force microscopy (AFM) [22,23]. Unfortunately, the imaging with these techniques requires labeling with fluorochromes for CLSM or fixation and complex dehydration processes for EM. Even the insightful visualization of FAs structure with AFM involves the de-roofing of fibroblasts and their subsequent fixation on the surface of glass coverslips [24]. Therefore, the subtle structures of FAs within the intact cells in physiologically relevant conditions have not yet been depicted.

This work aimed to visualize these faint cellular structures in their native hydrated form using X-ray microscopy, which is becoming an important imaging technique for cell morphology [25]. It was preceded by the preliminary identification of cytoskeleton structures and FAs with immunostaining and CLSM imaging. In the study, we have applied a well-known and very simple soft X-ray contact microscopy (SXCM) technique [26] using a newly developed laboratory setup based on a compact laser plasma source [27]. Several research groups have demonstrated SXCM as a suitable microscopy technique to visualize the biological object’s internal structures with high contrast and a nanometer spatial resolution [28,29,30,31,32,33,34,35,36,37]. In SXCM, the “water window” soft X-ray radiation in the wavelength range between 2.3 nm and 4.4 nm, corresponding to the absorption edges for oxygen and carbon, respectively, is used to obtain images of hydrated biological samples. The biomolecules in these spectral regions absorb photons at an order of magnitude stronger than water. This phenomenon depends proportionally on the thickness and function of biomolecules (according to the Beer-Lambert Law). The images at the nanoscale are obtained by recording an X-ray absorbance of the cells on the photoresist surface [26]. Thus, FA structures localized at the ventral side of the plasma membrane should be observable under SXCM. Bearing the above in mind, we used the recently developed SXCM system with a compact laser-plasma soft X-ray source [38] to visualize FAs within the cell membrane protrusions of native HCC38 breast cancer cells. Using this technique, we have demonstrated FA’s size and distribution in the cell under physiological conditions with nano-metric resolution.

## 2. Results

### 2.1. FAs Analysis Using CLSM

**Figure 1:**
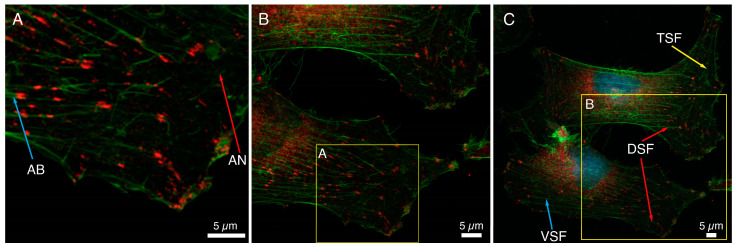
Analysis of the size and distribution of focal adhesion in HCC38 cells with immunostaining. (**A**–**C**) The focal adhesions in the cells were shown using anti-paxillin antibodies conjugated to Alexa Fluor 647 (red). The actin in the cytoskeleton was stained with phalloidin conjugated to Alexa Fluor 488 (green). Images under CLSM using Plan-Apochromat 63× objective at different scan area. Scale bar represents 5 μm.

The immunocytochemical staining of a basic FA protein, paxillin, and F-actin fibers in HCC38 cancer cells is shown in Figure 1. Numerous focal adhesions in lamellipodia and also in the proximity to the nucleus could be noticed. The association of FAs with the cytoskeleton of HCC38 cells was visualized, and different actin structures are shown in Figure 1. On the edge of the cell, loosely cross-linked structures of the actin network (AN, red arrow, Figure 1A) and parallel actin bundles (AB, blue arrow, Figure 1A), terminated with FAs and located closer to the cell’s center, were visible. When observing FAs at a larger scale, it can be noticed that FAs at the cell edge are smaller and less ordered than those observed closer to the cell center (Figure 1B). In that area, cross-linked actin fiber bundles formed thicker stress fibers (Figure 1C). Three types of stress fibers (SF): ventral SF, anchored at both ends by FAs (VSF, blue arrow), dorsal SF (DSF, red arrow), located near the front of the cell and attached by FAs only at one end, and finally transverse arcs (TSF, green arrow), arranged loosely without any connection to FAs, were visualized. The depicted FAs are mostly of elliptical shape and various sizes.

Figure 1 shows a large variation in the size of FAs determined with anti-paxillin antibodies; the median value of the area of FAs was 0.59 μm^2^. The 25th and 75th percentiles were 0.32 and 1.13 μm^2^, respectively. The median value of the diameters of FAs was equal to 0.88 μm (25th and 75th percentiles were 0.50 and 0.72 μm, respectively). This value was the average of the longer and shorter diameters of FAs, in which the median was 1.14 μm and 0.59 μm (25th and 75th percentiles were 0.87 and 1.42 for longer, 0.46 and 0.72 μm for shorter one, respectively). A double distribution of the diameters of FAs was shown on a histogram of the frequencies of diameters (Figure S1). The large dispersion of the FAs diameter, especially the longer one, could be observed.

### 2.2. FAs Analysis Using SXCM

The HCC38 cells on the photoresist’s surface absorbed X radiation (in the “water window” spectral range) to varying degrees, depending on the organic molecules (protein) density within the cells. The photoresist’s surface during radiation exposure was subjected to degradation at a different level, related to the efficiency of the radiation passage through cellular structures. After the detachment and removal of the cell debris and degraded fragments of photoresist (development), the imprint of the cells became visible. Due to the geometry of the system, assuming a source diameter of 0.5 mm and sample distance of 25 mm, the geometrical shadowing defining the spatial resolution is equal to x = h/50, where h is the distance of the object from the surface of the PMMA or the height of the object placed on top of the PMMA. For the object of h = 1 μm, the resolution is 20 nm; for h = 5 μm—the resolution is 100 nm. The geometrical shadowing is much larger than the diffraction-limited resolution $\frac{0.61\lambda }{NA}$, approximately a few nanometers, where λ is the illumination wavelength and NA is the numerical aperture of the optical system.

**Figure 2:**
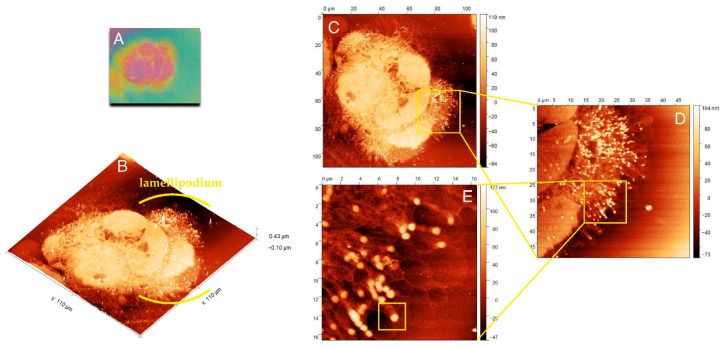
Morphology and internal structure of lamellipodium of HCC38 human breast cancer cells. (**A**) The preview image of HCC38 cells imprints obtained using AFM with the built-in 10× light-lens. AFM images of unfixed (**B**–**E**) HCC38 human breast cancer cells imprint after the exposure to X-rays in laser plasma-based SXCM. Topographies of the cell imprints performed in different scan size: (**B**,**C**) 110 µm × 110 µm; (**D**) 50 µm × 50 µm; (**E**) 16.5 µm × 16.5 µm. In the image (**E**) are marked single FA substructure. AFM images were processed using the Gwyddion 2.53 software.

Figure 2A shows the cell imprint observed under the light lens, a part of the AFM equipment. Scanning of this imprint with AFM allowed for a three-dimensional view of the internal structures of the cell, and Figure 2B presents the three-dimensional structure of actin and adhesion networks in the cell lamellipodium.

Figure 2C presents the whole cell with lamellipodium that was comprised of numerous actin fibers. The internal structure of lamellipodium at a higher magnification is shown in Figure 2D,E. These images presented a dendritic network of actin fibers terminated with FAs, clustered with a high density in the lamellipodium that pushed forward the cell. The highest density of FAs dots occurred in the front of the cell, but they could also be distinguished at its terminal part. Figure 2E shows that numerous FAs dots in HCC38 cancer cell.

### 2.3. FAs Structures Imaging Using CLSM vs. SXCM

**Figure 3:**
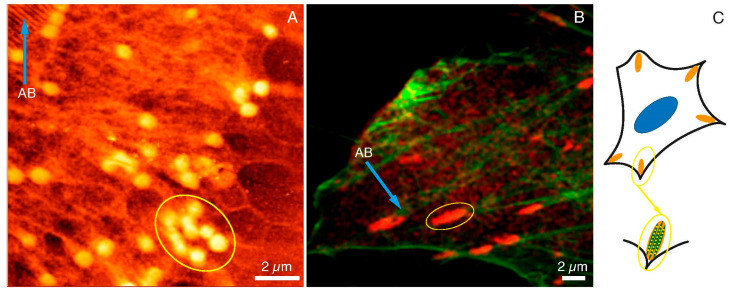
FAs and cytoskeleton structures under SXCM and CLSM. FAs (yellow ellipses) and actin bundles (blue arrows) as observed under (**A**) SXCM and (**B**) CLMS, (**C**) A diagram illustrating FAs structure and location within the cell. Scale bar represents 2 μm.

In Figure 3, the images of FAs and actin networks in HCC38 cancer cell under SXC (Figure 3A) and fluorescent (Figure 3B) microscopes were shown. The SXCM technique revealed FAs structures of more spherical shapes when compared to CLSM images where FAs were stained with anti-paxillin.

One has to bear in mind that fluorescent images were taken with the fixed dead cells, whereas SXCM images were recorded while cancer cells were still alive and attached to a substrate. Moreover, the resolution of SXCM was much higher than CLSM, which had a significant impact on the perception of adhesion substructures of the cell. The size of the agglomerates indicated in the SXCM image (yellow ellipse in Figure 3A) was very similar to the size of agglomerates specified in the CLSM image (yellow ellipse in Figure 3B). The lengths of the selected areas of SXCM and CLMS were 3.11 µm and 3.02 µm, respectively (data calculated with the ImageJ software). In the CLSM image, some spherical structures inside the marked area could also be noticed. These structures were not clearly visible due to the diffraction limit of the microscopic technique used. Figure 3A also shows the structures of the actin bundles (AB; blue arrow). They were also observed under the fluorescence microscope (AB; blue arrow in Figure 3B). Summarizing, we assumed that the single dot-like and fiber structures observed with SXCM are components of the internal packing of adhesive complexes. The images obtained with SXCM are of higher resolution and provide a more detailed view of these adhesive complexes’ internal substructures compared to CLSM. However, only immunostaining and recording the image with the fluorescent microscope could depict the identity of the proteins within the adhesion complexes. The draft in Figure 3C shows the arrangement of such dot-like substructures along the actin fibers in the FAs. Therefore, there is a need to apply both microscopic techniques to study FAs, and they are complementary to each other.

### 2.4. Internal Topographic Structure of FAs

**Figure 4:**
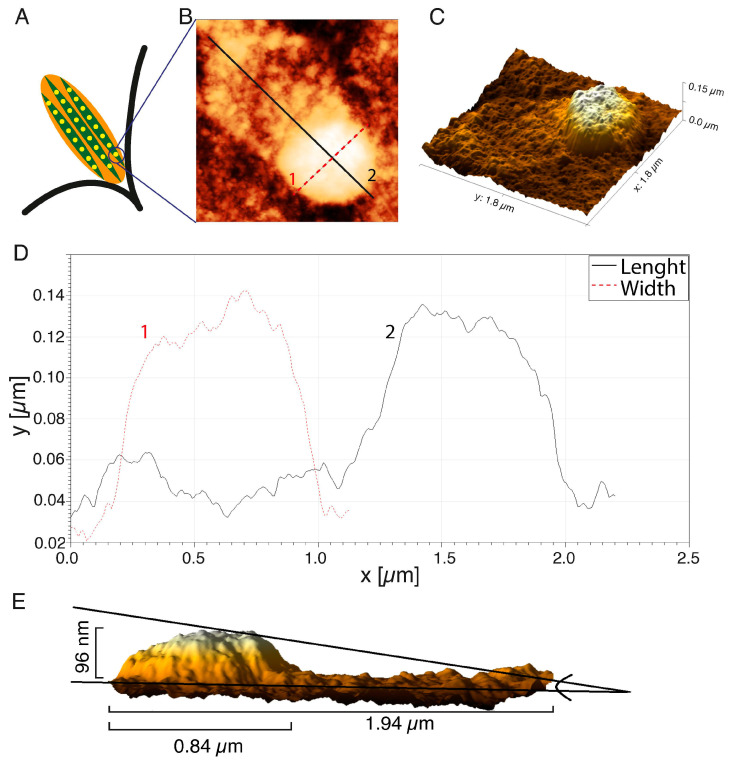
Analysis of topography of the single FA imprints: (**A**) scheme of the FA structure; (**B**) the AFM image of the FA; (**C**) the 3D reconstruction; (**D**) the height distribution of the FA imprints; (**E**) a side view of the FAs reconstruction. AFM images were processed using the Gwyddion 2.53 software.

The representative FA substructure topography was marked with a square in Figure 2E. The internal structure of one FAs is illustrated by the diagram in Figure 4A with the separated subunits. One subunit was visualized with AFM in Figure 4B.

**Figure 5:**
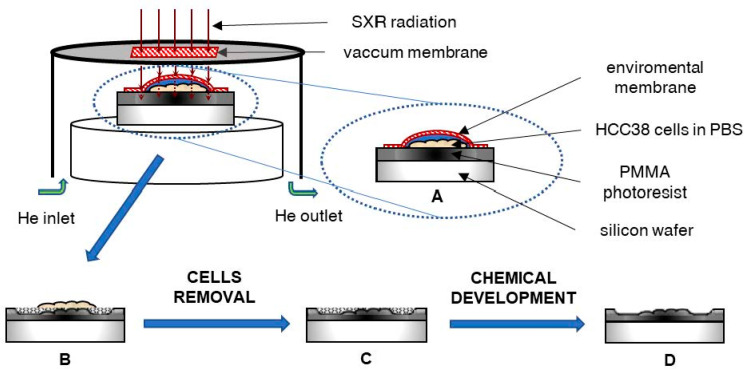
The scheme of the X-ray contact microscopy system: (**A**) The HCC38 cells attached to PMMA photoresist in PBS solution and covered with a membrane (protection against drying) were exposed to X-ray radiation. (**B**) The photoresist was degraded to an extent depending on the absorption of radiation by the cells. (**C**) Then, the cells were removed from the photoresist surface and (**D**) the photoresist was chemically developed to remove degraded polymer.

The 3D reconstruction of the AFM image (Figure 4C) showed some irregular distribution of organic molecule density within the structure. The highest density of organic molecules was at the terminal of the actin fiber, and this structure was rounded and resembled a dot. A protein “tail” was visible on the actin fiber (Figure 4B), which reflected an increase in the molecular density of the region of active actin polymerization or so-called “comet tail”—the region of the dynamic retrograde flux of the proteins from FA toward the center of the cell. The changes in the height of the FA dot (curve 1) and length, together with the “tail” area (curve 2), are presented in Figure 4D. The diameter of the FA’s dot was 0.84 µm, and the length along with the actin “tail” was 1.94 µm. A side view of a 3D reconstruction of the FA’s dot demonstrated that the height of this particular region increased to 96 nm. The height in the AFM image, however, does not directly represent the real height distribution of FAs, even though the photoresist operates in the linear regime (the photon exposure has a linear relation with the height of the photoresist). The absorption mechanism (exponential decay) in the material causes the relationship between the height of the structure, and the depth of the photons penetrating the PMMA is (theoretically) exp(-µd), where µ is the absorption coefficient of the material, and d is the thickness of the material. In order to get an average size of FA’s dot and tail, 30 FAs in the lamellipodium of cancer HCC38 breast cells were measured. The average height and diameter of the FA dot’s imprints were 91.0 ± 15.9 nm, and 0.84 ± 0.11 µm, respectively. The average length of FAs, together with the segment actin fibers with enhanced tail molecular density (as shown in Figure 5B, curve 2), was 1.56 ± 0.16 µm.

## 3. Discussion

The SXCM and CLSM techniques were used in this study to reveal the FAs size and localization in native living HCC38 cells. SXCM allows for the localization of specific structures within cells due to the concomitant absorption of soft X-ray radiation. The structures are identified by the difference in absorption coefficient between carbon-based structures and water/oxygen-rich material. Due to the differences in the absorption, the carbon-rich structures absorb more SXR radiation and appear taller in the AFM scan because fewer photons pass through such structures and expose (break the molecular bonds) less in the PMMA.

The great advantage of SXCM imaging is the possibility of observing the cell imprint at hydrated conditions without the necessity to use the subsequent procedures for fixation, immunostaining, and dehydration. The images of the HCC38 cell imprints were obtained with SXCM in PBS buffer and, therefore, in physiologically relevant conditions. SXCM allows for attaining images with high spatial resolution without complicated focusing optics required for other X-ray microscopy techniques. However, it should be mentioned here that observations of intracellular structures in living cells are also possible with interference reflection microscopy (IRM). This technique allows for studying the adhesion and mobility of living cells, but with a lower resolution [39,40] and in a cell-by-cell mode. This is in contrast to SXCM where the simultaneous recording of many cells is possible. In our opinion, this feature may be crucial in the analysis of dynamically reorganizing focal adhesions in cancer cells.

The detailed knowledge of cell adhesion and native FAs structure could bring new therapeutic options in combating cancer [41]. It holds especially true for TNBC treatment, since these cancer cells are resistant to endocrine therapies and do not have the known molecular targets for treatment [10,41]. The size and distribution of FAs determine the strength of adhesion, while the components of FAs activate downstream messengers and provide homeostasis of ECM-activated signaling pathways [11]. Moreover, the cell migration potential relies on FAs structure [42], and the rate of cell migration depends on the size of FAs [43]. An inverse relationship between cell speed and FAs size across various cell types occurs, and it has already been described in detail [44,45]. In this study, we calculated the median of the diameters of FAs as equal to 0.88 µm in HCC38 breast cancer cells. This finding stemmed from the observations under CLSM. Such a small size of FA was reported for cancers with high invasive potential, such as MDA231-M2 breast cancer cells. The average area of FAs in MDA231-M2 was ~0.9 µm^2^. This corresponds to our results, as this value fits between the 25th and 75th percentile (0.32—1.13 µm^2^) calculated for FAs of HCC38 cancer cells in ours study. An inhibitor of actin turnover, reversine, increased the area of FAs in MDA231-M2 cells two-fold and decreased the progression of breast cancer metastases [46].

SXCM allowed for demonstrating the internal spatial structure of the adhesion FAs complex in this study. The FAs were of small-diameter and round shape, while FAs under CLSM were bigger and oval. This smaller size of dots-like subunits visible under CLSM might result from visualization of only one protein in FAs complexes (i.e., paxillin) and prior fixation of the cells. The cell fixation alone may cause dehydration and lead actin fibers to stick together. It warrants a noticeable change in the resulting FA structure [24]. The size of FAs calculated from CLSM images displayed a greater dispersion of results than the size estimated from SXCM images. Paxillin is an adapter molecule involved in forming focal adhesions and can be found in both immature and mature FA multi-protein complexes that differ in size.

Imaging resolution of FAs by conventional fluorescence microscopy is insufficient to observe subunits throughout the FA structure. Studies using high-resolution fluorescence methods [47,48] support our observations on FAs composed of dots-like structures. Protein subunits are connected to the actin bundle and clustered in the so-called adhesive plate as shown in the diagram (Figure 4A). FAs are clustered into larger clusters, the size of which depends on the maturity of the adhesions.

In our imaging technique, a final step involved atomic force microscopy (AFM), which is not limited by the diffraction limit of optical microscopy. AFM has already been used to achieve super-resolution in focal adhesion imaging. Franz and Müller [24] have presented the AFM images of FAs; however, this was achieved after the de-roofing of REF52 fibroblasts using a short ultrasonic burst. This method allowed topographic observation of the FAs distribution with high resolution. However, the authors mentioned the short-term use of 2% glutaraldehyde and 4% paraformaldehyde to fix the cells, and it might have caused changes in the physical structure of FAs. They also mentioned a problem of tip contamination with cellular remains that might disturb AFM imaging. In addition, this method was used for topographic analysis of the surface of adhesion plates in cells and allowed for the observation of the arrangement of actin fibers and less of the FA “core” proteins [24]. The SXCM method used in this work did not have these shortcomings and allowed for obtaining FA’s imprints of the actin fibers network without any interference in the cell’s structure, i.e., without fixation or removing the cellular membrane.

However, this method requires optimization both in terms of the energy dose used during irradiation as well as the conditions of photoresist development on which the imprint of the cell is formed. Previous work [38] has shown that these conditions should be adapted to the specific cell line, as well as to the unique intracellular structures to be observed. The number of pulses for imaging the structures in the thin part of the HCC38 protrusions and the photoresist induction conditions were determined and used in this work when working with native living cells. Therefore, it was shown that similar conditions could be used for imaging fixed and native living cells using SXCM.

FAs interact with signals from outside and inside the cell comprised of transmembrane integrins and multiprotein complexes adhering to actin fibers and collectively link the intracellular cell cytoskeleton to the exterior ECM [49,50]. In this work, different actin fiber structures in the HCC38 cells using CLMS and SXCM methods were visualized. All three types of stress fibers were observed under CLSM. SXCM revealed the individual actin fibers instead, which were components of stress fibers; such regular and parallel structures have already been observed using AFM microscopy [51]. Therefore, the SXCM method seems to be a method that is complementary to CLSM in the visualization of stress fibers.

Another property of SXCM is generating 3D images of the internal cell structures because different cellular assemblies absorb X-ray radiation to varying degrees. This allowed for obtaining the 3D image of FAs with dot-like subunits attached to the actin fiber in this work. The dynamics of the internal structure of FA, as observed with high-resolution microscopy, is still a largely unexplored area [47]. Interactions of the individual FAs and actin networks are crucial in regulating FAs architecture and cell adhesion [50]. We plan to develop a new technique combining SXC microscopy with fluorescence microscopy. This would make it possible to determine the localization of fluorescently labeled FA proteins in a whole 3D cellular structure with a nanometer resolution of SXC microscopy. The combination of cryo-soft X-ray tomography (cryo-SXT) with cryo-fluorescence microscopy has already been used for the analysis of cellular substructures. Unfortunately, this technique required expensive equipment, including a synchrotron and cryo-preparation of cells before imaging [52,53,54]. The use of plasma to generate soft X-ray radiation might eliminate the need to use a synchrotron and freeze the sample. We assume that such correlative microscopy improves our understanding of FA organization in breast cancer cells.

## 4. Materials and Methods

### 4.1. Cell Culture

Human breast cancer cells HCC38 (ATCC^®^ CRL-2314TM) were cultured in RPMI-1640 medium with L-glutamine (Life Technologies, Grand Island, NY, USA) with 10% fetal bovine serum (FBS, Life Technologies, USA) and Antibiotic-Antimycotic (1% solution, Life Technologies, USA). The cells were maintained at 37 °C in a humidified atmosphere of 95% air and 5% CO_2_. The medium was replaced every three days. HCC38 breast cancer cells used in the experiments were from passage three.

### 4.2. Fluorescence Staining

Cells were fixed with 4% paraformaldehyde solution for 30 min and washed three times with PBS. Autofluorescence was quenched with 0.1M ammonium chloride (5′ RT). Cellular membranes were permeabilized with 0.5% Triton X-100 (30′ RT). Nonspecific binding sites were blocked by incubating the cells with the blocking buffer (5% *w/v* skimmed milk, 0.3% Triton-X100) for 1 h in RT. The cells were incubated with anti-paxillin rabbit antibody (1:250 in blocking buffer) overnight in 4 °C. After three washes with PBS, the cells were incubated with the blocking buffer containing secondary anti-rabbit antibody conjugated with Alexa Fluor 647 (1:500) and phalloidin conjugated with Alexa Fluor 488 (160 nM). Finally, cell nuclei were stained with DAPI (1 μg/mL), and the cover glass was mounted using ProLong™ Glass Antifade Mountant. Images of the cells were obtained on Zeiss Axio observer Z1 LSM 800 confocal microscope with Plan-Apochromat 63× objective. Fluorescence of Alexa Fluor 647, Alexa Fluor 488, and DAPI was acquired in separate channels with Ex/Em: 647/645–700 nm, Ex/Em: 488/476–620 nm, Ex/Em: 405/400–490 nm, respectively. The size of FAs was determined by counting the diameter of the 116 paxillin-positive objects using the ImageJ software (available at https://imagej.nih.gov, accessed on 24 May 2021), as shown before [55].

### 4.3. The Soft X-ray Contact Microscopy System

The soft X-ray contact microscopy system is based on a laser-plasma soft X-ray source, as described previously [27]. The total size of the system is 1.5 × 1.5 × 0.5 m^3^. The scheme of the image formation using the SXCM method is shown in Figure 5.

In the system, a plasma emitting soft X-ray radiation was produced due to irradiation of an argon/helium double stream gas puff target with a nanosecond Nd:YAG laser (HT 303, Ekspla, Lituania). Other SXR sources can also be used in SXCM, i.e., [56], providing enhancement in the photon emission; however, for the purpose of SXCM, the debris-free source based on a gas target has to be employed. The long-term use and constant SXR yield are mandatory for a precise photon flux, which it is necessary to deposit in the photoresist material. Such qualities in solid-state SXR sources, due to debris production and coating by the target material, are difficult to achieve. The conversion efficiency of the SXR gas puff-based source can be estimated from the ratio of E_p_/E_L_, where E_p_ is the energy from argon plasma excited by laser (13 mJ) and E_L_ is the energy of the driving laser (650 mJ). The energy conversion efficiency for the used SXCM system is 0.02 [57]. The plasma was produced in a vacuum chamber; however, hydrated samples were placed inside the sample chamber, which was filled with helium under a pressure of 1 bar at a constant flow of 0.5 L/min and mounted inside the vacuum chamber. The sample chamber was separated from the vacuum chamber with a 200 nm thick Si_3_N_4_ membrane (Silson, Warwickshire, UK) as a transmission window. For the SXCM imaging, HCC38 cancer cells were attached to the surface of PMMA photoresist, spin-coated on top of a Si wafer (ITME, Warsaw, Poland) and incubated for 24 h. The native cells were covered with an additional 70 nm thick Si_3_N_4_ membrane (to prevent the sample from drying out and also to keep cells alive in a helium environment) and exposed to 800 pulses of soft X-ray radiation in the wavelength range from about 3 nm (K-edge of nitrogen from Si_3_N_4_ membrane) to 4 nm. The photon flux was 112 ph/µm^2^ for one pulse, which gives 89.6 × 10^3^ ph/µm^2^ for 800 pulses interacting with the cells and the photoresist surface during exposure. After the exposure, the samples were rinsed with 5% sodium hypochlorite (NaClO, Achem, Wiązowna, Poland) for 5 min to remove cells from the photoresist’s surfaces. Then, the photoresists were developed using a mixture of methyl isobutyl ketone (Chempur, Piekary Śląskie, Poland) with isopropyl alcohol (POCH, Gliwice, Poland) for 4 min (ratio 1:2). The number of pulses during irradiation and the photoresist development conditions was optimized previously [38] and were adapted to the cell line and the type of observed structures. Finally, the samples were washed in pure isopropyl alcohol for 30 s and dried using compressed nitrogen. Cells imprints on the surfaces of the photoresist were visualized using AFM.

### 4.4. Atomic Force Microscopy

Soft X-ray contact images of HCC38 breast cancer cells were obtained by scanning the developed photoresists using atomic force microscopy (AFM, Nanoscope IV, Veeco, USA). The AFM measurements were carried out in a tapping mode with an anisotropic tip radius of 10 nm on rectangular cantilevers (MPP-11100, Veeco). Images were processed and analyzed using the Gwyddion 2.53 software (available at http://gwyddion.net/, accessed on 24 May 2021).

## Data Availability

Data is contained within the article or supplementary material.

## Supplementary Materials

The following are available online at https://www.mdpi.com/article/10.3390/ijms22147279/s1.
