# Supplementary material for: Adhesion of Triple-Negative Breast Cancer Cells under Fluorescent and Soft X-ray Contact Microscopy
Source: Int J Mol Sci. 2021 Jul 6;22(14):7279. doi: 10.3390/ijms22147279 (PMC8306697; doi:10.3390/ijms22147279)
Supplement: Supplementary file 1 [file ijms-22-07279-s001.zip › ijms-1263143-supplementary.pdf]

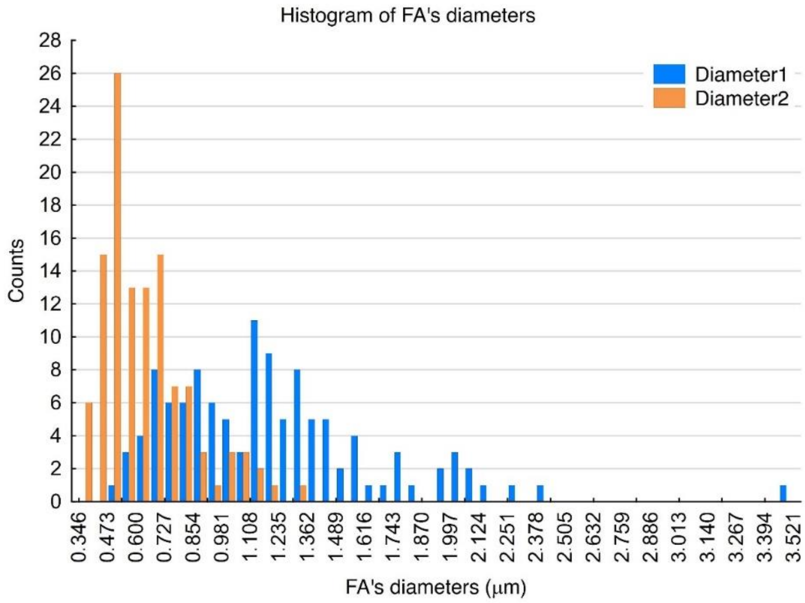

Figure S1. Histogram of the FAs' diameters in HCC38 breast cancer cells. The histogram shows the distribution of the longer (Diameter 1, blue) and shorter (Diameter 2, yellow) diameters of FAs stained with anti-paxillin antibodies. The total number of the objects counted was 116.
